# Fault diagnosis of aero-engines using transfer dispersion entropy and dispersion patterns

**DOI:** 10.1371/journal.pone.0348356

**Published:** 2026-05-27

**Authors:** Hong Zhang, Yin Zhang, Jingna Liu, Keqiang Dong

**Affiliations:** 1 Basic Courses Department, Tianjin Sino-German University of Applied Sciences, Tianjin, China; 2 College of Science, Civil Aviation University of China, Tianjin, China; Balikesir Universitesi, TÜRKIYE

## Abstract

Dispersion entropy (DE) can effectively detect the chaotic features in ordered sequences. However, DE is only defined by the probability distribution of static dispersion patterns, ignoring the dynamic transitions between pairwise dispersion patterns. Therefore, in this paper, by introducing the transition probability between pairwise dispersion patterns, we propose the transfer dispersion entropy (TDE) to detect the chaotic characteristics of the system. Additionally, based on both the difference in the number of each dispersion patterns and the difference in the transfer probability matrix, we define a dissimilarity measure for different sequences, namely transfer dissimilarity based on dispersion patterns (TDDP). Then, the proposed methods are verified on numerical simulation data and NASA-CMAPSS aero-engine simulation data.Compared with the existing entropy algorithms, the results show that TDE can not only capture more detailed chaotic changes, but also identify the early degradation of gas path components by detecting abnormal shifts in pattern transition behaviors, enabling more timely fault warnings.Finally, the combination of TDDP and multidimensional scaling can be used for similarity classification of aero-engine simulation time series.

## Introduction

When mechanical failures occur in equipment such as gearboxes and aircraft engines, the collected data tend to exhibit more non-stationary, aperiodic, and nonlinear characteristics. Accurately identifying fault-related information from the obtained time series is essential for timely equipment maintenance. Currently, several advanced signal processing methods are employed in fault detection for large-scale machinery due to their ability to effectively analyze dynamic information within time series. For instance, Huo et al. [1] applied wavelet transform to decompose vibration signals into multiple layers and then utilized a particle swarm optimization algorithm to determine the optimal parameters of a pulse-based model, achieving promising fault diagnosis results. Liu et al. [2] demonstrated how time¨Cfrequency domain analysis can be used for fault detection in tandem systems: variational mode decomposition was first applied to extract characteristic frequency bands from current signals, after which the Shannon entropy of these band signals was calculated to assess signal complexity. Mohsen and El-Yazeed [3] adopted an autoregressive moving average model to preprocess circuit responses under different fault conditions, deriving a set of features to train and test a backpropagation neural network for fault classification. However, these signal processing approaches often rely on empirical or prior knowledge [4] and are associated with high computational complexity. Moreover, they typically require the predefinition of numerous parameters that are difficult to select appropriately.

Owing to their reliance on fewer parameters, independence from prior knowledge, and straightforward applicability without pretreatment, entropy-based approaches have been widely adopted in fault diagnosis [5]. In their work [6], Huo et al. provide a thorough review of the theoretical development of several foundational entropy methods, clarifying their interrelations. They further outline representative applications of entropy in mechanical fault detection. As a measure of system complexity, approximate entropy (AE) [7] is utilized to quantify repetitive transients in machine condition monitoring [8]. The introduction of sample entropy (SE) [9], which assesses series complexity by computing the proportion of newly generated patterns, has led to a broader application of entropy theory in fault diagnosis [10,11]. Fuzzy entropy (FE) [12], as a modified version of SE, incorporates fuzzy theory to compute state probabilities, thereby achieving greater stability and demonstrating reliable performance in bearing fault classification [13]. Despite their utility as fault detection tools, these methods possess certain limitations. SE exhibits significant variability and lacks continuity [14]. Moreover, when processing long data series, both AE and FE suffer from lower computational efficiency [15]. To address these issues, Bandt and Pompe [16] proposed permutation entropy (PE), which evaluates dynamic complexity through ordinal permutation and offers a new perspective for mechanical fault detection [17]. Recognizing that PE does not account for amplitude differences, Rostaghi and Azami introduced dispersion entropy (DE) [18], an irregularity measure that incorporates amplitude relationships. DE is less sensitive to sudden signal changes and can effectively identify chaotic features within ordered sequences, making it applicable in fields such as simulation and diagnosis [19,20].

Meanwhile, nonlinear dynamic indicators such as Lempel-Ziv complexity (LZC) have been integrated with symbolic patterns. Li et al.have proposed two notable LZC-based indicators: the multivariate threshold-adjusted permutation LZC (MTPELZC) [21] to enhance sensitivity to permutation patterns in multivariate systems, and the multiscale similarity fuzzy LZC (MSFLZC) [22] to preserve correlation information between dispersion patterns. However, both methods have limitations: MTPELZC requires complex parameter selection for multivariate settings and suffers from high computational cost; MSFLZC is sensitive to similarity tolerance and also computationally expensive. More importantly, neither models the dynamic transition patterns between successive states, which is crucial for capturing system evolution.

In recent years, integrating transition probabilities to capture the dynamic evolution of time series has become an important branch of entropy measure research. For example, Zhang et al. [23] introduced the transition probability between adjacent permutations into PE to quantify the complexity of time series. A novel method termed symbolic transition entropy [24] was proposed to measure the dynamic complexity of multi-step transitions in time series, demonstrating better performance in machinery health monitoring [25]. Along this line, a related research direction has focused on distance metrics derived from transition probability matrices in different domains. Xu et al. developed a time-frequency dual-domain prediction (TFDDP) model, which employs self-supervised learning to align time-domain and frequency-domain representations for fault diagnosis with limited labeled data [26]. He et al. proposed a time-frequency dual-domain contrast and fusion (TFDDCF) model, which introduces contrastive learning to enhance feature discrimination under scarce labeled conditions [27]. These models, however, operate within a deep learning framework that relies on data augmentation and contrastive loss functions, whereas the present work introduces a purely entropy-based distance metric that retains full interpretability without requiring labeled training data. The complementary nature of these two lines of inquiry is noteworthy: deep learning models excel at learning high-dimensional feature representations from large datasets, while entropy-based methods offer physically interpretable, parameter-light alternatives that can serve as front-end feature extractors or as independent diagnostic tools. Notably, these models focus on fuzzy membership and distance fusion, lacking a unified entropy framework or a corresponding transfer entropy indicator to characterize system complexity, which distinguishes them from our physics-based, lightweight, and interpretable TDE and TDDP.

Despite these advances, existing studies exhibit a significant theoretical limitation: the transition probability framework has primarily been applied to describe the evolution of ordinal relationships, but has not yet been systematically extended to analyze dynamic transitions in amplitude distribution. Inspired by these developments, this paper combines the transition probability between pairwise dispersion patterns with the dispersion entropy algorithm, further accounting for the dynamic transformation across different dispersion patterns. Compared with TPE, TDE not only captures sequential pattern changes but also incorporates amplitude-level transition information, which may enhance sensitivity to amplitude modulation and impact characteristics commonly present in mechanical fault signals. Relative to basic DE, TDE introduces memory effects through a Markov chain model, revealing the dynamic influence of the current state on future states and thereby capturing richer system dynamic information. This method not only exhibits stronger parameter stability compared to existing entropy algorithms but also detects more subtle chaotic variations. Moreover, based on both the quantitative differences in dispersion patterns and the divergence in transition probability matrices, we define a metric to quantify the discrepancy between different sequences or systems. Finally, the feasibility and superiority of TDE and TDDP are verified on numerical simulation data and NASA-CMAPSS aero-engine simulation data, a standard benchmark dataset in this field, before summarizing the main contributions of this article as follows.

(1) A complexity metric, transfer dispersion entropy (TDE), is proposed by introducing the dynamic transfer between pairwise dispersion patterns into dispersion entropy.
(2) A distance metric for different time series, transfer dissimilarity based on dispersion patterns (TDDP), is defined based on the static quantity distribution of dispersion patterns and the probability distribution of dynamic transitions between pairwise dispersion patterns. Combined with multidimensional scaling (MDS) method, the TDDP results are visualized.
(3) The parameter settings of TDE are canvassed. Moreover, the performance of TDE is assessed from aspects such as detectability of dynamic changes, consistency with Lyapunov exponents and robustness to parameters. The comparative experiments demonstrate that the proposed methods have better discernibility than other entropy measures or distance measures.
(4) The feasibility and superiority of TDE and TDDP in mechanical fault detection are once more verified with the collected normal datasets of aircraft engines and datasets containing faults.

The TDE and TDDP proposed in this paper are physical features with strong interpretability, which are complementary to data-driven fault diagnosis methods such as deep learning and digital twins. For example, methods based on Graph Convolutional Networks (GCN) can effectively model the topological relationships between sensors [28], while the method proposed in this paper can serve as a front-end feature extractor, converting high-dimensional non-stationary original signals into low-dimensional indicators with clear physical meanings. It can be independently used for fault monitoring and classification, and can also be input into deep learning models as high-quality features to improve their performance, providing a new basic component for hybrid intelligent diagnosis systems that integrate physical features and data-driven models.

The rest of this article is organized as follows. Methodologies section provides the definition and specific calculation steps of the methods proposed in this article. Simulation experiments section analyzes and evaluates the performance of TDE and TDDP by simulation experiments. Experimental validation section further applies the methods to aircraft engine data, proving the feasibility of them. Finally, Conclusion section summarizes this article.

## Methodologies

### Dispersion entropy

Unlike the permutation entropy, the dispersion entropy (DE) considers the magnitude relationship between amplitudes. For a sequence $x=\{x_{j},j=1,2,\ldots ,N\}$, the calculation of DE mainly includes the following six steps.

Step 1. Normalization. The sequence x is mapped to the sequence $y=\{y_{j},j=1,2,\ldots ,N\}$ through the normal distribution function,


$$\begin{array}{c} y_{j}=\frac{1}{\sigma \sqrt{2\pi }}\int_{-\infty }^{x_{j}}e^{-\frac{(t-\mu {)}^{2}}{2\sigma^{2}}}dt \end{array}$$ (1)

where $y_{j}\in (0,1)$, μ and σ are the mean and standard deviation of time series x, respectively.

Step 2. Linear transformation. Every $y_{j}(j=1,2,\ldots ,N)$ mapped to a number within the set {1,2,…,c} by a linear transformation,


$$\begin{array}{c} z_{j}^{c}=\text{round}(c\cdot y_{j}+0.5) \end{array}$$ (2)

i.e., the sequence *y* is mapped to a sequence *z*^*c*^ containing *c* classes, where *c* is an integer.

Step 3. Phase space reconstruction. According to the embedding dimension *m* and time delay τ, the sequence *z*^*c*^ is embedded into N−(m−1)τ subsequences. Each subsequence is represented as


$$\begin{array}{c} Z_{j}=\{z_{j}^{c},z_{j+\tau }^{c},\ldots ,z_{j+(m-1)\tau }^{c}\},\;j=1,2,\ldots ,N-(m-1)\tau . \end{array}$$

Step 4. Map each subsequence $Z_{j}\;(j=1,2,\ldots ,N-(m-1)\tau )$ to a corresponding dispersion pattern $\Pi_{j}=\pi_{v_{0}v_{1}\ldots v_{m-1}}$ (abbreviated as $\pi_{\left(v\right)}$), where $v_{0}=z_{j}^{c}$, $v_{1}=z_{j+\tau }^{c}$, ..., $v_{m-1}=z_{j+(m-1)\tau }^{c}$. Then, generate the dispersion pattern sequence ${\left\{\Pi_{j}\right\}}_{j=1}^{N-(m-1)\tau }$. There are *c*^*m*^ types of possible dispersion patterns, namely, $v=1,2,\ldots ,c^{m}$, because each pattern consists of *m* elements, and each element can be any integer between 1 and *c*.

Step 5. For each type of dispersion pattern $\pi_{\left(v\right)}=\pi_{v_{0}v_{1}\ldots v_{m-1}}\;(v=1,2,\ldots ,c^{m})$, calculate its probability by the following equation,


$$\begin{array}{c} P\left(\pi_{v_{0}v_{1}\ldots v_{m-1}}\right)=\frac{\text{Count}(\pi_{v_{0}v_{1}\ldots v_{m-1}})}{N-(m-1)\tau }, \end{array}$$ (3)

where Count($\pi_{v_{0}v_{1}\ldots v_{m-1}}$) represents the number of the pattern $\pi_{v_{0}v_{1}\ldots v_{m-1}}$ among all dispersion patterns. Therefore, the probability matrix for all dispersion patterns can be denoted as,


$$\begin{array}{c} \Psi =\left[\;p(\pi_{\left(1\right)})\;p(\pi_{\left(2\right)})\;\cdots \;p(\pi_{\left(c^{m}\right)})\;\right] \end{array}$$ (4)

Step 6. Connecting to the definition of Shannon entropy, the DE of sequence *x* is defined as,


$$\begin{array}{c} \begin{array}{cc} DE(x,m,c,\tau )\;\;\; & =-\sum_{\pi_{v_{0}v_{1}\ldots v_{m-1}}}p\left(\pi_{v_{0}v_{1}\ldots v_{m-1}}\right)\log p\left(\pi_{v_{0}v_{1}\ldots v_{m-1}}\right)\\ & =-\sum_{v=1}^{c^{m}}p(\pi_{\left(v\right)})\log p(\pi_{\left(v\right)}). \end{array} \end{array}$$ (5)

From the above calculation steps, it can be seen that the parameters *m*, *c* and τ need to be set up in advance. According to the recommendations of relevant literature [29–31], we usually set *m* = 2, τ=1,c∈[4,8]. When all possible dispersion patterns occur with equal probability, the maximum value $\log c^{m}$ of DE is obtained. Therefore, the normalized dispersion entropy (nDE) is,


$$\begin{array}{c} nDE(x,m,c,\tau )=\frac{DE(x,m,c,\tau )}{\log c^{m}}. \end{array}$$ (6)

### Transfer dispersion entropy

Conventional dispersion entropy (DE) is defined primarily based on the static probability distribution of dispersion patterns, and its core limitation is that it cannot characterize the dynamic transition behaviors among different patterns. To address this drawback, this paper proposes transfer dispersion entropy (TDE). Notably, TDE is fundamentally different from the existing transfer permutation entropy (TPE) in its symbolization basis: TPE relies on ordinal information, whereas TDE is constructed on amplitude information. Specifically, the first four computational steps of TDE are consistent with those of DE. In its critical fifth step, TDE not only computes the distribution probabilities of static dispersion patterns, but also further introduces the transition probabilities between dispersion patterns, so as to accurately model the dynamic characteristics of the system.

Quantifying the pattern transition in the dispersion pattern sequence is the key to feature extraction. Here, the Markov chain theory is used to describe the transitions between dispersion patterns. The dispersion pattern sequence is regarded as a Markov chain, the dispersion pattern transition is regarded as a one-step Markov process [32]. We predominantly concentrate on the relationship between the current state and the next state [33], i.e., the transfer behavior between the current dispersion pattern $\prod_{j}$ (assuming that $\prod_{j}\pi (a)$) and the next dispersion pattern $\prod_{j+1}$(assuming that $\prod_{j+1}\pi (b)$). Then, the one-step transfer probability from π(a) to π(b) is,


$$\begin{array}{c} P_{\pi (a)\to \pi (b)}=\frac{\text{Count}\{\Pi_{j}:j\leq N-(m-1)\tau ,\;\Pi_{j}=\pi (a),\;\Pi_{j+1}=\pi (b)\}}{\text{Count}\{\Pi_{j}:j\leq N-(m-1)\tau ,\;\Pi_{j}=\pi (a)\}} \end{array}$$ (7)

where $a,b\in \{1,2,\ldots ,c^{m}\}$. In fact, the transfer probability $\pi_{\left(a\right)}\to \pi_{\left(b\right)}$ is the conditional probability of two adjacent dispersion patterns, namely, $p(\pi_{\left(b\right)}|\pi_{\left(a\right)})$. Based on the above formula, we can obtain the transfer probability matrix,


$$\begin{array}{c} \Phi =[\begin{array}{cccc} P_{\pi_{\left(1\right)}\to \pi_{\left(1\right)}} & P_{\pi_{\left(1\right)}\to \pi_{\left(2\right)}} & \cdots & P_{\pi_{\left(1\right)}\to \pi_{\left(c^{m}\right)}}\\ P_{\pi_{\left(2\right)}\to \pi_{\left(1\right)}} & P_{\pi_{\left(2\right)}\to \pi_{\left(2\right)}} & \cdots & P_{\pi_{\left(2\right)}\to \pi_{\left(c^{m}\right)}}\\ \vdots & \vdots & \ddots & \vdots \\ P_{\pi_{\left(c^{m}\right)}\to \pi_{\left(1\right)}} & P_{\pi_{\left(c^{m}\right)}\to \pi_{\left(2\right)}} & \cdots & P_{\pi_{\left(c^{m}\right)}\to \pi_{\left(c^{m}\right)}} \end{array}] \end{array}$$ (8)

The jth row of the matrix ϕ represents the probability distribution of all possible patterns at the next moment when the current dispersion pattern is *pi*(*j*). According to the properties of probability distribution, $\forall a,b\in \{1,2,\ldots ,c^{m}\},\;p_{\pi (a)\to \pi (b)}\geq 0,\;\forall a\in \{1,2,\ldots ,c^{m}\},\;\sum_{b=1}^{c^{m}}p_{\pi (a)\to \pi (b)}=1$. Finally, combined with the concept of joint entropy, based on the probability distribution of the dispersion patterns and the transfer probabilities between adjacent dispersion patterns, the TDE of the time series *x* is obtained as,


$$\begin{array}{c} TDE(x,m,c,\tau )=-\sum_{a=1}^{c^{m}}\sum_{b=1}^{c^{m}}\left(p(\pi_{\left(a\right)})p_{\pi_{\left(a\right)}\to \pi_{\left(b\right)}}\right)\log\left(p(\pi_{\left(a\right)})p_{\pi_{\left(a\right)}\to \pi_{\left(b\right)}}\right). \end{array}$$ (9)

When all possible dispersion patterns $\pi_{\left(v\right)}\;(v=1,2,\ldots ,c^{m})$ have the same probability $\frac{1}{c^{m}}$, and all possible pattern transitions $\pi_{\left(a\right)}\to \pi_{\left(b\right)}\;(a=1,2,\ldots ,c^{m},\;b=1,2,\ldots ,c^{m})$ also have the same probability $\frac{1}{c^{m}}$, TDE has the maximum value $\log c^{2m}$. Therefore, the normalized TDE (nTDE) is,


$$\begin{array}{c} nTDE(x,m,c,\tau )=\frac{TDE(x,m,c,\tau )}{\log c^{2m}} \end{array}$$ (10)

Equation (9) can be expressed by matrices as,


$$\begin{array}{c} \text{TDE}(𝐱,𝐦,𝐜,\tau )=-\sum_{a=1}^{c^{m}}\sum_{b=1}^{c^{m}}{\left[(\Psi ⊗\Phi^{T})⊙\log(\Psi ⊗\Phi^{T})\right]}_{ba} \end{array}$$ (11)

where ⊗ represents the Khatri-Rao product, ⊙ is the Hadamard product, [·]_*ba*_ denotes the element in the bth row and the ath column of a matrix.

### Transfer dissimilarity based on dispersion patterns

The TDE proposed in Transfer dispersion entropy section is a measure to evaluate the complexity of a single time series. In order to measure the differences between different sequences or systems, we define a distance measure between two time series, called transfer dissimilarity based on dispersion patterns (TDDP). Its specific calculation steps are as follows.

Step 1. For two time series $x=\{x_{j},j=1,2,\ldots ,N\}$, $y=\{y_{j},j=1,2,\ldots ,N\}$, according to the calculation of TDE, we can obtain two number sequences $\{\text{Count}(\pi_{\left(v\right)}){\}}_{v=1}^{c^{m}}$ and $\{\text{Count}(\pi_{\left(v\right)}^{\prime }){\}}_{v=1}^{c^{m}}$ of all possible dispersion patterns, two transition probability matrices ϕ and $\phi^{\prime }$, respectively.

Step 2. The dissimilarity between two transition probability matrices ϕ and $\phi^{\prime }$ is measured by calculating the Frobenius norm of the difference ϕ – $\phi^{\prime }$ between the two matrices, i.e.,


$$\begin{array}{c} \sqrt{\sum_{i=1}^{c^{m}}\sum_{j=1}^{c^{m}}\omega_{ij}^{2}} \end{array}$$ (12)

where $\omega_{ij}$ represents the elements in the matrix ϕ – $\phi^{\prime }$ with *c*^*m*^ rows and *c*^*m*^ columns. Step 3. To comprehensively quantify the discrepancy in the static information of sequences *x* and *y*, the Euclidean distance between two number sequences of dispersion patterns $\{\text{Count}(\pi_{\left(v\right)}){\}}_{v=1}^{c^{m}}$ and $\{\text{Count}(\pi_{\left(v\right)}^{\prime }){\}}_{v=1}^{c^{m}}$ is calculated as,


$$\begin{array}{c} \sqrt{\sum_{v=1}^{c^{m}}{\left(\text{Count}(\pi_{\left(v\right)})-\text{Count}(\pi_{\left(v\right)}^{\prime })\right)}^{2}} \end{array}$$ (13)

In summary, TDDP measures the dissimilarity between two time series from two respects, respectively, the divergence of dynamic transitions between two adjacent patterns in the dispersion pattern sequences and the difference in the number of each dispersion pattern. A smaller value of TDDP indicates a higher similarity between two time series.

### Multidimensional scaling

Multidimensional scaling (MDS) is a multivariate data analysis technique in machine learning that embeds high-dimensional data into a low-dimensional space [34,35]. Given an nn similarity (or distance) matrix, it addresses the problem of finding a reasonable low-dimensional representation for n objects, which can be intuitively visualized in a graphical form. In such a visualization, each point in the low-dimensional space stands for an object, while the distances between these points are highly correlated with the similarities between the corresponding objects. That is, the more similar two objects are, the closer the two corresponding points in low-dimensional space are.

To verify whether the proposed TDDP can accurately quantify the relationships among different time series, we apply MDS to visualize the TDDP matrix and compare TDDP with other distance measures. As a versatile tool, MDS covers a wide range of variants, which can be classified into metric MDS and non-metric MDS according to the measurement scale of similarity (or distance) data. Metric MDS refers to the form where the original similarity or distance values are directly employed as interval-scale measurements. In this paper, we select the classic and most commonly used metric MDS, which takes the Euclidean distance as the distance metric in the low-dimensional subspace projected from the original data.

## Simulation experiments

### Simulation data

To evidence the performance of TDE, several typical nonlinear chaotic systems [36] are used in this study, which are classified into three types, including non-inverted maps (such as the logistic map, the sine map and the cubic map), dissipative maps (such as Hénon map), conservative maps (such as Lorenz map). The detailed information of five maps are as follows.

(i) Logistic map
  $$\begin{array}{c} x_{n+1}=rx_{n}(1-x_{n}). \end{array}$$
  Parameter selection:*r* = 3.8,initial condition:*x*_1_ = 0.4. The one-dimensional logistic map is in a completely chaotic state at 3.57<r≤4,the trajectory of the equation in this interval exhibits chaotic making it a classic example for studying chaotic phenomena.
(ii) Sine map
  $$\begin{array}{c} x_{n+1}=r\sin\left(f_{0}\right)\left(\pi x_{n}\right) \end{array}$$
  Parameter selection: *r* = 0.98, initial condition: *x*_1_ = 0.4. When r∈(0.87,0.93) and (0.95, 1), the sine map exhibits the chaotic phenomena.
(iii) Cubic map
  $$\begin{array}{c} x_{n+1}=ax_{n}^{3}-bx_{n} \end{array}$$
  Parameter selection: *a* = 1, *b* = 2.5, initial condition: *x*_1_ = 0.4. The sequences generated by cubic map are chaotic when b∈(2.3,3).
(iv) Hénon map
  $$\begin{array}{c} x_{n+1}=1-ax_{n}^{2}+y_{n} \end{array}$$
  $$\begin{array}{c} y_{n+1}=bx_{n} \end{array}$$
  Parameter selection: *a* = 1.4, *b* = 0.3, initial condition: *x*_1_ = 0.1, *y*_1_ = 0.2. The research results indicate that when a∈[1.07,1.4] and *b* = 0.3, chaotic attractors exist in the two-dimensional Hénon map, the generated chaotic sequences have strong randomness.
(v) Lorenz map
  $$\begin{array}{cc} \dot{x} & =a(y-x),\\ \dot{y} & =(r-z)x-y,\\ \dot{z} & =xy-bz. \end{array}$$
  Parameter selection: *a* = 10, *r* = 28, *b* = 83, initial condition: *x*_1_ = 10, *y*_1_ = 1, *z*_1_ = 0. Lorenz map is a three-dimensional nonlinear dynamic system with attractors. Under the condition that a and b maintain unchanged, Lorenz map enters the chaotic state when *r* > 24.74.

### Simulation experiments on the TDE

**Fig 1:**
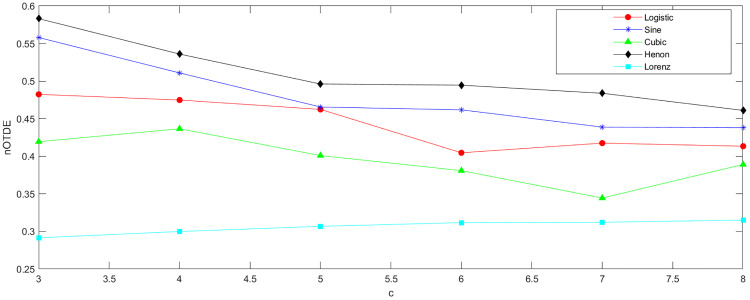
The nTDE of sequences generated by five chaotic maps under different values of *c.*

Parameter c denotes the number of symbol classes used when transforming the original sequence into a symbolic sequence. If c is set too small, data points with large amplitude differences may be classified into the same category, whereas an excessively large value of c can make TDE sensitive to noise and result in a substantial growth in computational time. To explore the influence of parameter c, we first carry out experiments with the five chaotic maps described in Simulation data section, where c is varied from 4 to 8. The sequences generated by these maps have a length of 8000, with the time delay τ=1 and embedding dimension *m* = 2. We then calculate the nTDE values of the five sequences, as presented in Fig 1. For the effective discrimination of these sequences, it is advisable to set *c* = 6 or *c* = 7.

**Fig 2:**
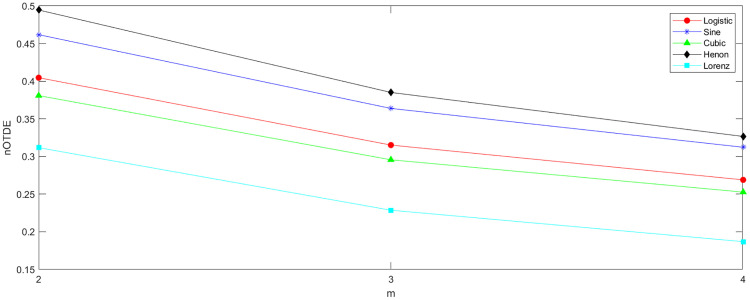
The nTDE of sequences generated by five chaotic maps under different embedding dimensions *m.*

As for the embedding dimension *m*, similar to the DE algorithm, the total number of possible dispersion patterns *c*^*m*^ should not exceed the length of the sequence. Furthermore, an excessively large value of *m* should be avoided, as it may result in the loss of important dynamic information. To investigate the effect of parameter *m* on the TDE, we still employ the above five chaotic sequences with a length of 8000 as experimental data, where *c = 6,* τ=1, and m is set within the range of [2, 8]. The corresponding results are presented in Fig 2. It can be observed that when m = 2, the nTDE values not only clearly distinguish the differences among the five chaotic sequences but also consume the least computational time. Therefore, we recommend setting the parameter m to 2 in the TDE method.

**Fig 3:**
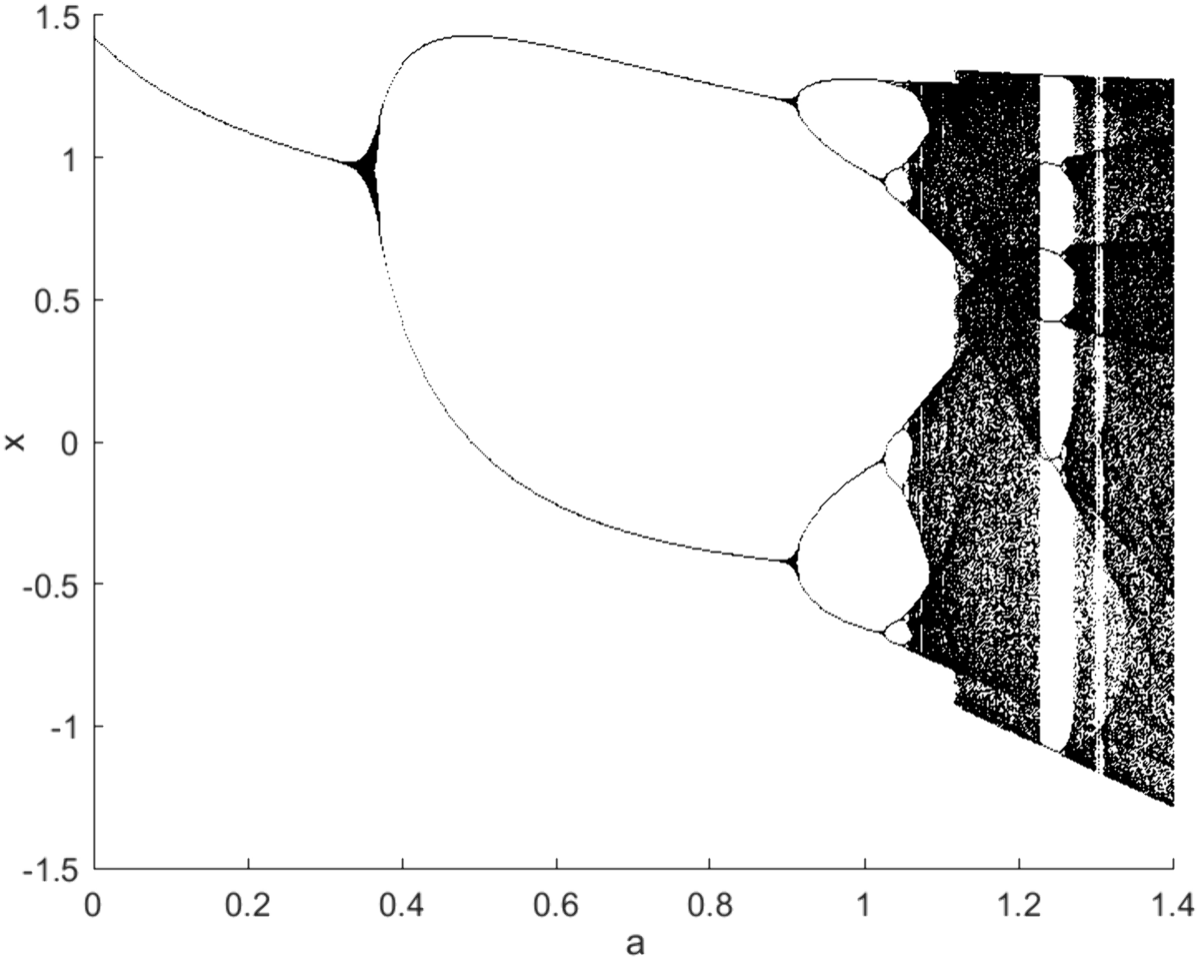
Bifurcation diagram of the Hénon map. *a* is the parameter in the Hénon map.

**Fig 4:**
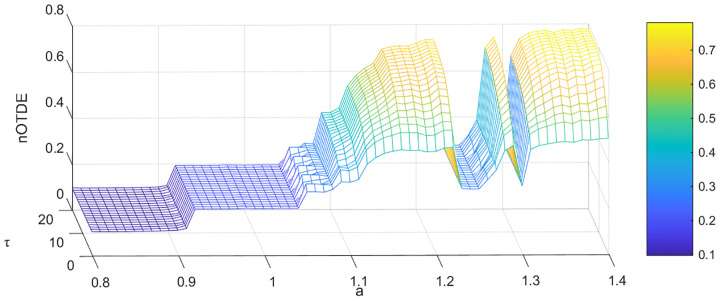
The nTDE of Hénon map. *a* is the parameter in the Hénon map, τ is the time delay.

Because the change of the calculation amount caused by the increase of time delay τ is much smaller than that caused by the increase of embedding dimension *m*, by extending the range of time delay, we can obtain a more intuitive three-dimensional graph to further understand the properties of the system. Next, we select the Hénon map, where the parameter a ranging from 0.8 to 1.4 with step 0.01 and *b* = 0.3. For a∈[0.8,1.07], the sequences generated by Hénon map are almost all periodic sequences, but the period size is different. When a∈[1.07,1.4], the whole system is mainly in a chaotic state, however, some specific values of a still make the system non-chaotic and have periodic results. The bifurcation diagram of the Hénon map is shown in Fig 3. The nTDE, a and τ are drawn into a three-dimensional graph, as shown in Fig 4. In Fig 4, the nTDE values of periodic sequences are very small and almost unaffected by the increase of time delay. However, with the increase of τ, the nTDE values of chaotic sequences first increase continuously and then gradually approach a stable value. It is worth noting that when a∈[1.07,1.4], there are two locations where the nTDE values diminish considerably, namely, the blue regions sandwiched between the yellow regions. We find that it is consistent with the bifurcation diagram of the Hénon map in Fig 3. Thus, we can draw the conclusion that the chaotic series and the periodic series can be identified based on the nTDE and time delay τ.

**Fig 5:**
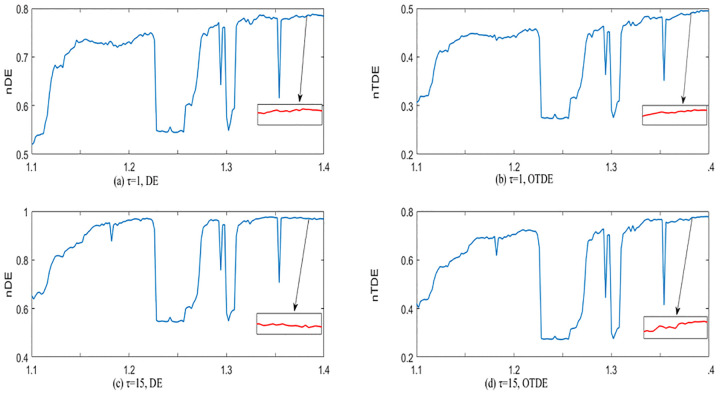
(a) DE of the Hénon map when *τ* = 1. (b) TDE of the Hénon map when *τ* = 1. (c) DE of the Hénon map when *τ* = 15. (d) TDE of the Hénon map when *τ* = 15.

**Fig 6:**
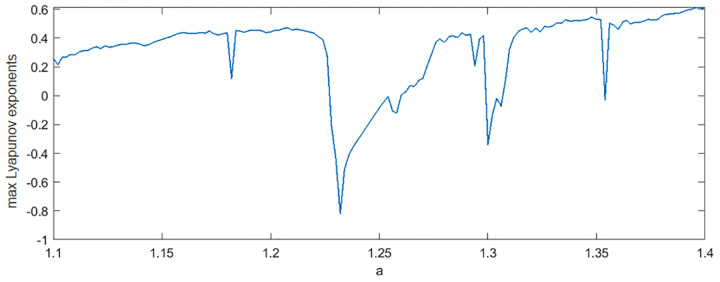
The Lyapunov exponent graph of Hénon map.

In order to better explain the influence of time delay τ on TDE, we compare TDE with DE under different τ, where *m* = 2 and *c* = 6 are selected. The range of a in Hénon map is set to [1.1,1.4] and the step size is 0.002. We normalize and locally amplify them to further show that nTDE is more stable with respect to time delay than nDE. The results are shown in Fig 5. Although the curves of nDE and nTDE are similar when τ=1, when τ=15, the nDE curve shows a decreasing trend in a∈[1.36,1.4], while nTDE still increases gradually, and the nTDE curve is very consistent with the Lyapunov exponent [37] in Fig 6. For a multidimensional nonlinear system, if the Lyapunov characteristic exponent is greater than zero, it means that the system is in a chaotic state. The larger the exponent is, the more obvious the chaotic characteristics are. In fact, it can be seen from the bifurcation diagram in Fig 3 that the sequences in a∈[1.36,1.4] are more and more chaotic, so the performance of TDE is more stable in distinguishing chaotic properties under different time delay. Additionally, under different τ, the TDE curves have the same performance, TDE can describe the chaotic characteristics of chaotic systems in both cases. Because the information in nonlinear systems can be considered more fully when τ=1, we suggest that the time delay τ in TDE is 1.

**Fig 7:**
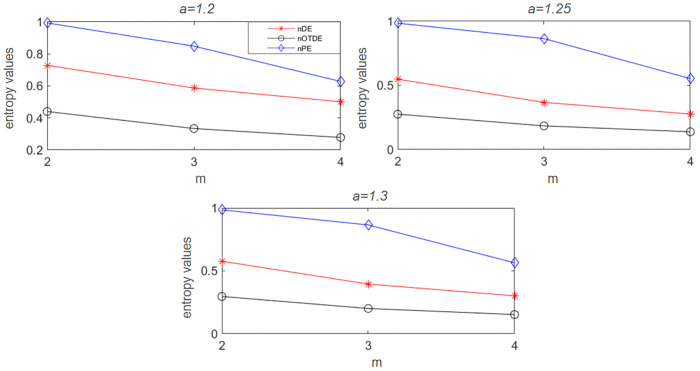
The entropy results of Hénon map with *a* = 1.2, 1.25 and 1.3 respectively under different embedding dimensions *m* = 2, 3, 4.

**Fig 8:**
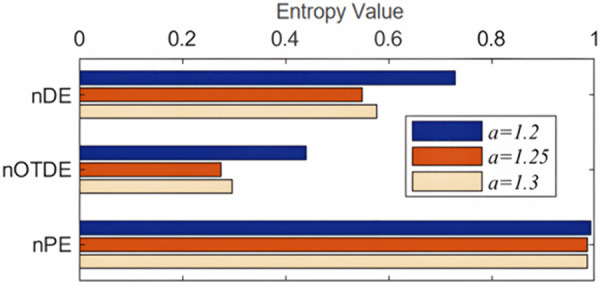
The distinguishing ability of nDE, nTDE and nPE for different chaotic sequences generated by the Hénon map with *a* = 1.2, 1.25 and 1.3 when *m* = 2.

To further illustrate the superiority of TDE for the embedding dimension *m*, under different values of *m*, we also calculate the TDE, DE and permutation entropy (PE) of three sequences generated by Hénon map with *a* = 1.2, 1.25, 1.3, where m=2,3,4,τ=1. In Fig 7, it can be found that three different sequences have consistent entropy results, i.e., compared with DE and PE, TDE is less affected by the increase of embedding dimension, indicating that TDE is less dependent on the parameter *m*. Fig 8 more intuitively shows the distinguishing ability of three entropy methods for different chaotic sequences when *m* = 2. The TDE and DE results of three sequences have obvious differences, while PE has the worst performance. In general, the experiment shows that TDE has certain advantages over DE and PE.

### Robustness analysis of TDE to noise

In practical fault diagnosis scenarios, sensor signals are often contaminated by noise, which may impair the performance of entropy-based feature extraction methods. To evaluate the noise resilience of the proposed TDE, we perform a comparative robustness analysis between TDE, DE, and PE under different levels of additive Gaussian white noise. We first generate a chaotic sequence of length *N* = 4000 using the Hénon map (*a* = 1.3, *b* = 0.3), to which additive Gaussian white noise is introduced across a signal-to-noise ratio (SNR) range of −10 dB to 20 dB. For each SNR value, the experiment is repeated 100 times to ensure statistical significance, while the entropy parameters are set as *m* = 4, *c* = 6 and τ=1. The number of amplitude classes *nc* = 6, which matches the feature extraction requirements in practical fault diagnosis scenarios.

**Fig 9:**
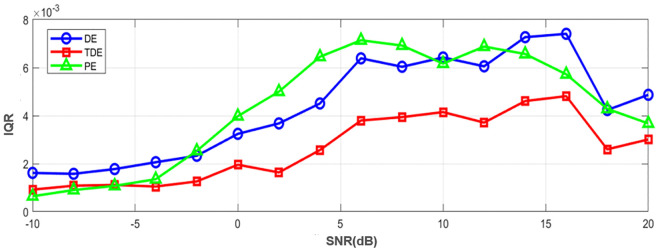
Stability comparison of DE, TDE and PE under different signal-to-noise ratios (measured by IQR).

The robustness is quantified using the coefficient of interquartile range (IQR). As shown in Fig 9. Across the entire signal-to-noise ratio (SNR) range, the interquartile range (IQR) of TDE is consistently lower than that of DE and PE. This indicates that transfer dispersion entropy (TDE) exhibits the best performance in terms of signal stability and noise robustness, with particularly prominent advantages in low-SNR environments. This is of great significance for noise-prone scenarios such as mechanical fault diagnosis.

### Simulation experiments on the TDDP

**Fig 10:**
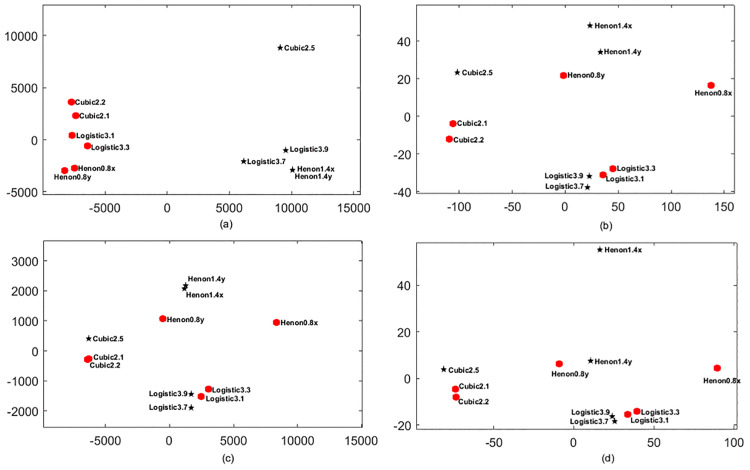
Two-dimensional MDS results of 11 sequences. **The red circles represent periodic sequences, the black circles represent chaotic sequences. (a)** MDS result of TDDP. **(b)** MDS result of standard Euclidean distance. **(c)** MDS result of Manhattan distance. **(d)** MDS result of Chebyshev distance.

In this section, we mainly verify whether TDDP can effectively quantify the discrepancies between different time series. Since relying merely on direct numerical results cannot fully reflect the practical performance of TDDP, we employ MDS to visualize the results obtained by TDDP. We further compare TDDP with the standard Euclidean distance, Manhattan distance and Chebyshev distance, and the corresponding results of these three distance measures are also visualized via MDS. We adopt the logistic map (*r* = 3.1, 3.3, 3.7, 3.9), cubic map (*b* = 2.1, 2.2, 2.5) and Hénon map (including *x* and *y* components, *a* = 0.8, 1.4 to generate 6 periodic sequences and 5 chaotic sequences. With the parameters set as *c* = 6, *m* = 2 and τ=1, we calculate the TDDP values between any two sequences by the proposed TDDP method, and finally obtain an 1111 TDDP matrix. In this work, we use the actual values of all the computed dissimilarities, so that the TDDP matrix is visualized by metric MDS. The visualization results of the four metrics are illustrated in Fig 10.

As can be seen from Fig 10, four measures have diverse characteristics. The MDS results of the standard Euclidean distance, Manhattan distance and Euclidean distance are somewhat similar. The logistic map, the cubic map and the Hénon map can only be roughly classified in Fig 10(b), 10(c) and 10(d). There is no obvious boundary between 6 periodic sequences and 5 chaotic sequences. However, the MDS results of TDDP show that three types of chaotic systems are divided into two different regions. In Fig 10(a), periodic sequences can be well distinguished from chaotic sequences, which means TDDP has better separability for different categories of sequences. In addition, TDDP has better clustering performance for the same category of sequences, i.e., whether in the region of periodic sequences or in the region of chaotic sequences, the sequences generated by the logistic map, the cubic map and the Hénon map are well clustered together, respectively. The experimental results show that TDDP has the advantage of measuring the dissimilarity of different time series from the perspective of chaotic characteristics, and it has good separability for chaotic features. This is because TDDP is defined based on the number of each dispersion pattern in the DE and the transition probability between adjacent dispersion patterns in the TDE, moreover, DE and TDE can effectively distinguish the chaotic characteristics of sequences.

## Experimental validation

In this section, the main purpose is to verify the feasibility and superiority of TDE in mechanical fault diagnosis, as well as the good performance of TDDP in the classification of real time series.

### Data description

Aircraft vibration faults are more frequent, especially when the aircraft has been in continuous service for about 6 years. There are many reasons for aircraft shaking, in addition to weather factors, the aircraft engine is also one of the main causes of vibration. Engine failure or performance degradation is usually related to multiple factors such as a decrease in low-pressure rotor speed (N1) and high-pressure rotor speed (N2), an increase in exhaust temperature (EGT) and fuel flow (WF), and the increased intake pressure. Therefore, the engine fault analysis is often carried out by studying the sequences of its gas path parameters N1, N2, EGT and WF [36].

**Fig 11:**
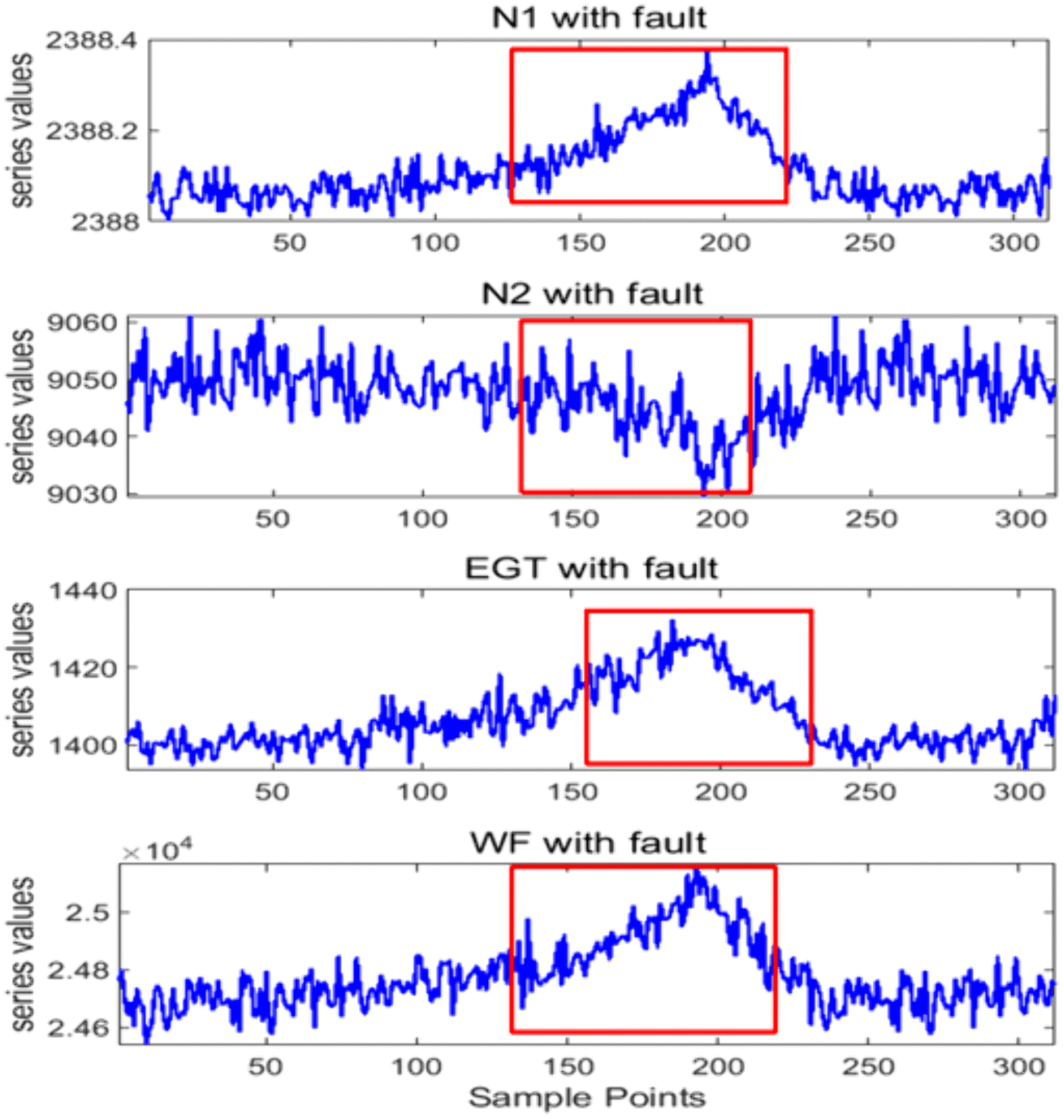
N1, N2, EGT and WF data with faults during the cruise phase from the NASA-CMAPSS aero-engine simulation dataset. The red boxes indicate the faulty parts.

To explore the application of TDE in aero-engine fault diagnosis, we adopt the NASA-CMAPSS aero-engine simulation data (FD001 subset). As a widely recognized standard simulation dataset for aero-engine health management, it is generated by component-level engine models. Since real aero-engine operating data is difficult to be publicly available due to engineering confidentiality, this dataset is employed to verify the proposed method. Four gas path parameters (N1, N2, EGT, WF) under fault conditions in the cruise phase are selected for analysis, as shown in Fig 11. It is seen that they have different fault characteristics. During the malfunction, the rotor speeds N1 and N2 are lower than normal values, while EGT and WF increase significantly. After the fault is resolved, they gradually return to a stable state and maintain normal operation.(data source: https://data.nasa.gov/dataset/cmapss-jet-engine-simulated-data).

**Fig 12:**
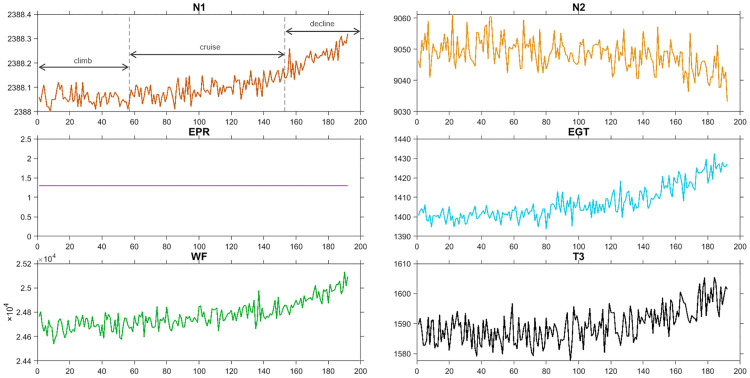
Normal six gas path parameters in the climb, cruise, and descent stages from the NASA-CMAPSS aero-engine simulation dataset.

In addition, Fig 12 shows the time series of six gas path parameters under normal operating conditions, generated from the same NASA-CMAPSS simulation benchmark, covering the climb, cruise, and descent stages, which are adopted to verify the effectiveness of TDDP on different operating conditions. As shown in Fig 12, N1, WF, EGT, and T3 exhibit clear upward trends during the decline phase, while N2 remains relatively stable with minor fluctuations, and EPR stays nearly constant throughout all phases. These distinct temporal patterns across different operating stages provide a realistic basis for evaluating the TDDP method’s ability to capture sequential similarities and classify time series data effectively.

### Fault detection based on TDE

**Fig 13:**
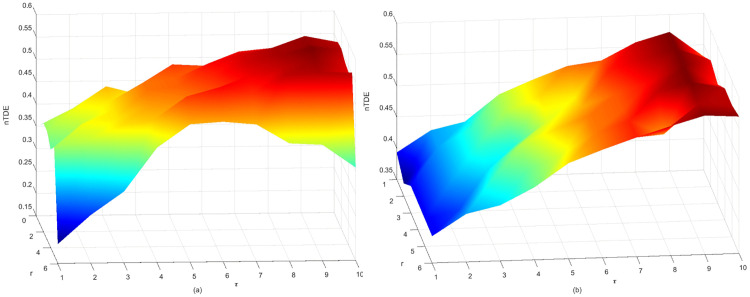
The nTDE of WF sequence in the cruise phase. **(a)** The fault WF sequence. **(b)** The normal WF sequence. τ is the time delay and *r* is the sliding window.

**Fig 14:**
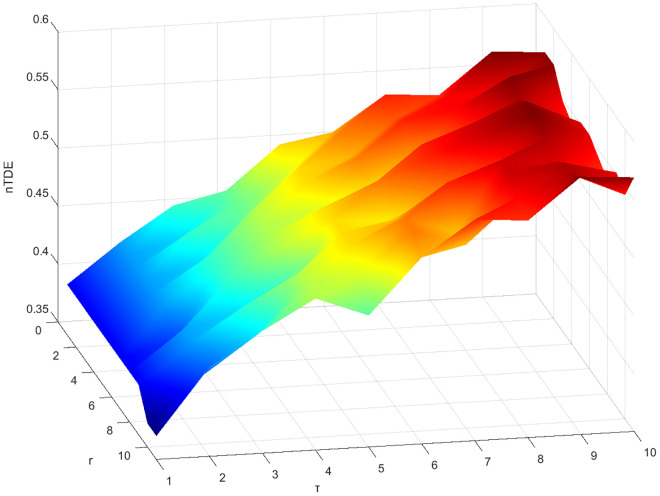
The nTDE of the normal WF sequence in three stages. τ is the time delay and *r* is the sliding window.

In the simulation experiments, we have demonstrated that extending the range of the time delay τ can affect the TDE results, while the corresponding three-dimensional graph enables effective distinction between periodic sequences and chaotic sequences. Subsequently, we carry out experiments using the fault WF data and normal WF data in the cruise phase as examples, where the time delay τ is set to [1,20], the sliding window size *d* = 30, and the sliding step is fixed at 15. In addition, we calculate the nTDE values of the normal WF sequence across three stages, with a sliding step of 15 to maintain consistency with the cruise phase analysis. The corresponding results are shown in Figs 13 and 14.

In Fig 13, the nTDE values of the fault WF sequence (a) exhibit a more pronounced fluctuation and higher overall amplitude compared to the normal WF sequence (b). This distinct difference can be utilized to identify the timing of aircraft engine malfunctions and locate the anomalous segments within the gas path parameter sequences. In addition, the results in Fig 14 show that the TDE of the normal WF sequence exhibits different distribution rules in the climb, cruise and decline stages, indicating that the combination of TDE and time delay τ can distinguish different flight stages. The experiments on NASA-CMAPSS aero-engine simulation gas path parameters further validate that TDE can effectively characterize the dynamic changes of simulated fault signals.

**Fig 15:**
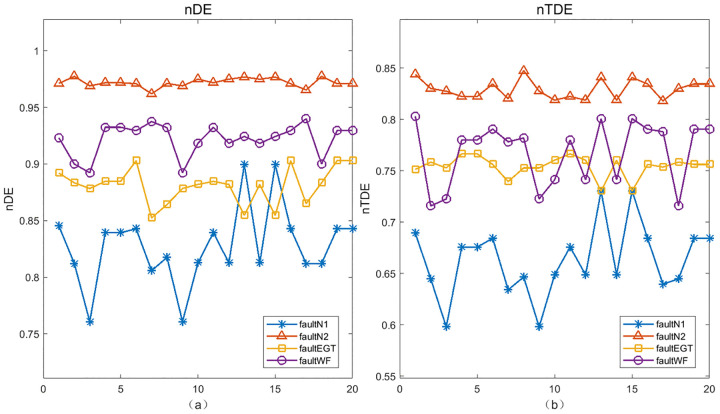
Distribution diagrams of different fault sequences. (a) nDE. (b) nTDE.

We also compare the performance of nTDE and nDE by applying the series of four gas path parameters N1, N2, EGT and WF Fig 11. We randomly select 20 groups of samples from the fault data by setting random initial points, and each sample length is 80. The discrimination effect of normalized nDE and normalized nTDE on different fault sequences is studied respectively. As can be seen from Fig 15, TDE can distinguish the four fault sequences with high clarity,where each fault sequence shows a distinct and non-overlapping trend in the nTDE distribution diagram.In the DE distribution diagram, faultN1 sequence is easily confused with faultN2, and faultEGT and faultWF also exhibit significant overlap, leading to poor discriminability between different fault types. Therefore, nTDE distinguishes different fault sequences more clearly and consistently than nDE. It should be noted that longer series can display the information more comprehensively. However, in this experiment, precise and repeatable discrimination results are still obtained when the sample length is 80, demonstrating the remarkable applicability and robustness of nTDE even for relatively short fault sequences.

**Fig 16:**
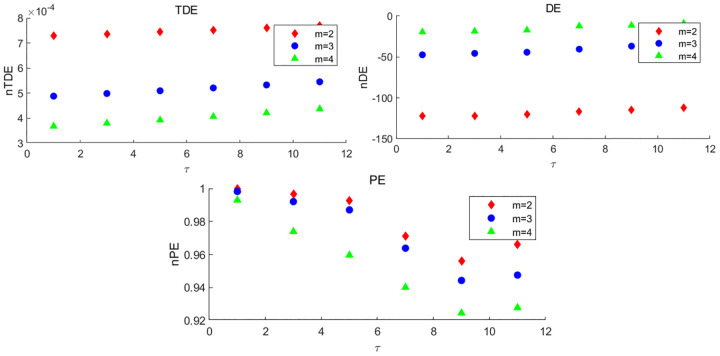
The joint effects of embedding dimension m and time delay τ on the TDE, DE and PE.

Finally, we take the real WF data in Fig 12 as an example to further verify that TDE has more stable performance than DE and PE when the parameter changes. In Fig 16, we simultaneously consider the combined effects of the embedding dimension *m* and the time delay τ on TDE, DE and PE. The results show that when the time delay is fixed to any value, the change of embedding dimension has a significant quantitative impact on PE and DE. However, no matter what the value of τ is, TDE is relatively stable with the increase of *m*, maintaining nTDE within a narrow range of $\left[3.5\times 10^{-4},7.5\times 10^{-4}\right]$, meaning that TDE is less affected by the embedding dimension.

Similarly, when the embedding dimension remains unchanged and the time delay τ continues to increase, we can draw analogous conclusions that TDE has better stability than DE and PE under different values of τ. For DE, nDE fluctuates noticeably with τ, while PE’s nPE decreases monotonically from 0.99 to 0.96 with increasing τ. In contrast, TDE’s nTDE only shows a slight upward trend with τ, staying within a stable range across all tested τ values.We know that when reconstructing a time series, an overly large τ leads to uncorrelated delay coordinates, while an overly small τ causes data redundancy, making the selection of τ critical [38]. Due to the smaller dependence of TDE on both embedding dimension *m* and time delay τ, the TDE method has stronger robustness than DE and PE.

### Classification performance comparison

To comprehensively evaluate the discriminative power of different entropy-based features on NASA-CMAPSS aero-engine simulation data, we conduct a comparative study involving four entropy measures: the proposed Transfer Dispersion Entropy (TDE), conventional Dispersion Entropy (DE), Permutation Entropy (PE), and Transfer Permutation Entropy (TPE). The engine gas path parameter data, specifically the fuel flow (WF) sequences, are utilized for this analysis. Both normal operation data and simulated fault data are processed using a sliding window approach with a window size of 25 and step size of 5, generating *N* = 156 samples. Each subsequence is characterized by the four entropy features calculated with parameters *m* = 4, *c* = 6, and τ=2. A Radial Basis Function (RBF) Support Vector Machine (SVM) classifier is employed for binary classification (normal vs.faulty), with the dataset partitioned into 70% training and 30% testing sets, and hyperparameters optimized via 5-fold cross-validation.

**Fig 17:**
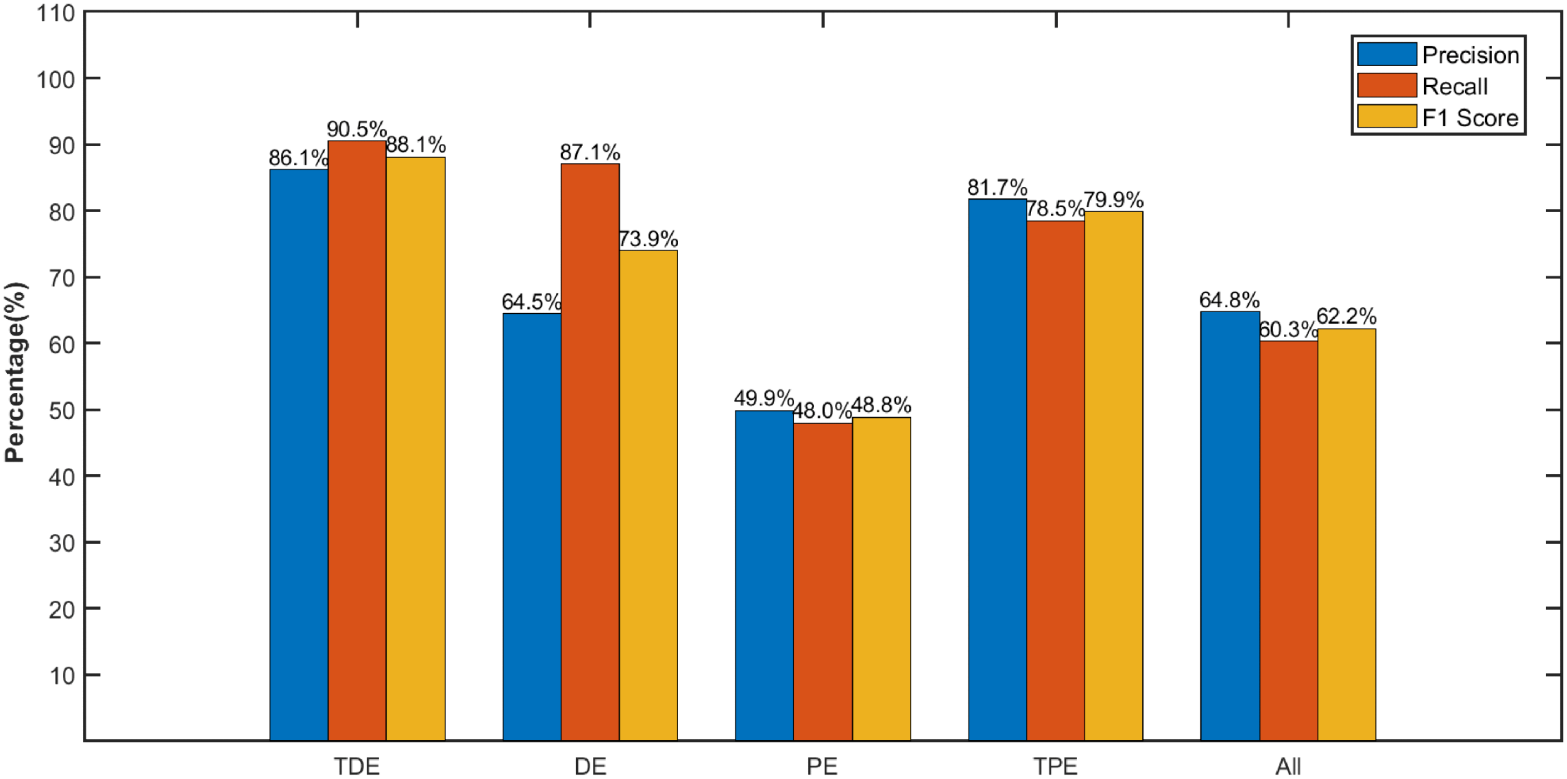
Performance assessment of different fault diagnosis approaches using precision, recall and F1 Score.

As shown Fig 17, the four entropy features exhibit distinct performance characteristics in aero-engine fault diagnosis. TDE performs best across core metrics:achieving a precision of 86.1%, recall of 90.5%, and F1-score of 88.1%, demonstrating its advantage in balancing fault detection accuracy and reducing false alarms. Although PE achieves a recall rate of 48.0% (detecting partial faults), its precision is only 49.9%, leading to a high false alarm rate and poor overall F1-score. TPE performs moderately with a precision of 81.7% but it is still outperformed by TDE. DE shows balanced but suboptimal performance. Notably, the combination of all features yields does not outperform the single TDE feature. This indicates that TDE already contains the most effective discriminative information for WF data fault diagnosis, and redundant feature fusion even degrades overall performance.

### Similarity classification based on TDDP and MDS

**Fig 18:**
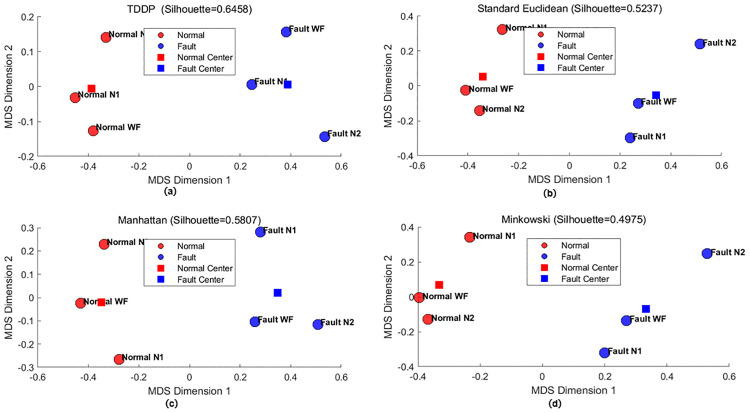
MDS Visualization and silhouette coefficient evaluation of fault and normal sequences with various distance measures. **(a)** TDDP. **(b)** Standard Euclidean distance. **(c)** Manhattan distance. **(d)** Minkowski distance.

To verify the effectiveness of TDDP in temporal category discrimination, we selected three normal and three fault sequences from the NASA-CMAPSS aero-engine simulation dataset, with 50 data points in each sequence, all under the cruise condition, and compared TDDP with the standard Euclidean distance, Manhattan distance and Chebyshev distance via MDS visualization (Fig 18). Results show that TDDP achieves a silhouette coefficient of 0.6458 with clear separation between normal and fault samples, while the three traditional distances show lower coefficients (0.5237 for Euclidean, 0.5807 for Manhattan, 0.4975 for Minkowski)and severe sample overlap. By integrating static distribution entropy (DE) and dynamic transfer distribution entropy (TDE) information, TDDP outperforms traditional distance metrics in capturing the temporal characteristics of engine gas path data, demonstrating superior discriminability and suitability for engine fault diagnosis.

## Conclusion

As an improved entropy measure, dispersion entropy exhibits notable advantages in identifying chaotic characteristics. In this paper, we propose transfer dispersion entropy (TDE) by introducing the dynamic transition behavior between consecutive dispersion patterns into the original dispersion entropy framework. TDE incorporates not only the static distribution information of dispersion patterns but also the dynamic transfer characteristics among them. Furthermore, we define a novel distance metric for diverse time series, namely transfer dissimilarity based on dispersion patterns (TDDP), which is constructed by combining the quantitative discrepancies of dispersion patterns and the differences in their corresponding transfer probability matrices. In the simulation experiments, TDE is compared with other entropy approaches to validate its superiority in characterizing the chaotic properties of time series. Meanwhile, in comparison with conventional distance metrics, the combination of TDDP and multidimensional scaling (MDS) is proven to achieve better separation and classification between chaotic and periodic sequences. Finally, experimental validations are carried out on numerical simulation data and NASA-CMAPSS aero-engine simulation data, which verify that TDE and TDDP are effective for aero-engine simulation fault detection and time series classification. Using these real-world datasets, we further confirm that the integration of TDDP and MDS enables more accurate similarity classification of different sequences.

### Limitations and future work

Although the effectiveness of TDE and TDDP has been verified on numerical simulation data and NASA-CMAPSS aero-engine simulation data, several limitations still exist, which also point out future research directions. First, although experimental analyses have yielded recommended parameter settings, a generalized adaptive optimization strategy applicable to diverse practical scenarios remains to be developed. Second, the proposed TDE is currently limited to univariate time series. In practical aero-engine systems, multiple sensors are adopted to collect correlated signals. Therefore, extending the proposed method to multivariate transfer dispersion entropy (MTDE) to capture the coupled dynamic characteristics of multi-channel signals is an important direction for future research. Third, all aero-engine experiments in this study are based on the NASA-CMAPSS simulation benchmark due to the confidentiality and inaccessibility of real engine data. Future work will focus on verifying the proposed method on actual measured aero-engine data if available, so as to further improve engineering practicability. In addition, as mentioned in the introduction, the proposed TDE and TDDP can be used as effective physical feature extractors. Therefore, the in-depth integration of the proposed method with advanced deep learning models to construct hybrid end-to-end intelligent fault diagnosis frameworks is also a promising research direction.
